# Autosomal Recessive Dilated Cardiomyopathy due to *DOLK* Mutations Results from Abnormal Dystroglycan O-Mannosylation

**DOI:** 10.1371/journal.pgen.1002427

**Published:** 2011-12-29

**Authors:** Dirk J. Lefeber, Arjan P. M. de Brouwer, Eva Morava, Moniek Riemersma, Janneke H. M. Schuurs-Hoeijmakers, Birgit Absmanner, Kiek Verrijp, Willem M. R. van den Akker, Karin Huijben, Gerry Steenbergen, Jeroen van Reeuwijk, Adam Jozwiak, Nili Zucker, Avraham Lorber, Martin Lammens, Carlos Knopf, Hans van Bokhoven, Stephanie Grünewald, Ludwig Lehle, Livia Kapusta, Hanna Mandel, Ron A. Wevers

**Affiliations:** 1 Department of Neurology, Institute for Genetic and Metabolic Disease, Radboud University Nijmegen Medical Centre, Nijmegen, The Netherlands; 2 Department of Laboratory Medicine, Institute for Genetic and Metabolic Disease, Radboud University Nijmegen Medical Centre, Nijmegen, The Netherlands; 3 Department of Human Genetics, Nijmegen Centre for Molecular Life Sciences, Donders Institute for Brain, Cognition, and Behaviour, Nijmegen, The Netherlands; 4 Department of Pediatrics, Institute for Genetic and Metabolic Disease, Radboud University Nijmegen Medical Centre, Nijmegen, The Netherlands; 5 Department of Cell Biology and Plant Biochemistry, University of Regensburg, Regensburg, Germany; 6 Department of Pathology, Radboud University Nijmegen Medical Centre, Nijmegen, The Netherlands; 7 Institute of Biochemistry and Biophysics, Polish Academy of Sciences, Warsaw, Poland; 8 Heart Institute, Schneider Children's Medical Centre of Israel, Petach Tikwa, Israel; 9 Metabolic Unit and Unit of Pediatric Cardiology, Meyer Children's Hospital, Rambam Medical Center, Technion–Israel Institute of Technology, Haifa, Israel; 10 Great Ormond Street Hospital, UCL Institute of Child Health, University College London, London, United Kingdom; 11 Pediatric Cardiology Unit, Edith Wolfson Medical Center, Holon, Israel; The Jackson Laboratory, United States of America

## Abstract

Genetic causes for autosomal recessive forms of dilated cardiomyopathy (DCM) are only rarely identified, although they are thought to contribute considerably to sudden cardiac death and heart failure, especially in young children. Here, we describe 11 young patients (5–13 years) with a predominant presentation of dilated cardiomyopathy (DCM). Metabolic investigations showed deficient protein N-glycosylation, leading to a diagnosis of Congenital Disorders of Glycosylation (CDG). Homozygosity mapping in the consanguineous families showed a locus with two known genes in the N-glycosylation pathway. In all individuals, pathogenic mutations were identified in *DOLK*, encoding the dolichol kinase responsible for formation of dolichol-phosphate. Enzyme analysis in patients' fibroblasts confirmed a dolichol kinase deficiency in all families. In comparison with the generally multisystem presentation in CDG, the nonsyndromic DCM in several individuals was remarkable. Investigation of other dolichol-phosphate dependent glycosylation pathways in biopsied heart tissue indicated reduced O-mannosylation of alpha-dystroglycan with concomitant functional loss of its laminin-binding capacity, which has been linked to DCM. We thus identified a combined deficiency of protein N-glycosylation and alpha-dystroglycan O-mannosylation in patients with nonsyndromic DCM due to autosomal recessive *DOLK* mutations.

## Author Summary

Idiopathic dilated cardiomyopathy (DCM) is estimated to be of genetic origin in 20%–48% of the patients. Almost all currently known genetic defects show dominant inheritance, although especially in younger children recessive causes have been proposed to contribute considerably to DCM. Knowledge of the genetic causes and pathophysiological mechanisms is essential for prognosis and treatment. Here, we studied several individual young patients (5–13 years old) with idiopathic and sometimes asymptomatic dilated cardiomyopathy. The key to identification of the gene was the finding of abnormal protein N-glycosylation. Via homozygosity mapping and functional knowledge of the N-glycosylation pathway, the causative gene could be identified as dolichol kinase (*DOLK*). Since DCM is very rare in N-glycosylation disorders (Congenital Disorders of Glycosylation, CDG) and most patients with CDG present with a multisystem involvement, we studied the underlying pathophysiological cause of this life-threatening disease. Biochemical experiments in affected heart tissue showed deficient O-mannosylation of alpha-dystroglycan, which could be correlated with the dilated cardiomyopathy. Our results thus highlight nonsyndromic DCM as a novel presentation of DOLK-CDG, via deficient O-mannosylation of alpha-dystroglycan.

## Introduction

Dilated cardiomyopathy (DCM) is a life-threatening disease characterized by left ventricular enlargement and systolic dysfunction, which can lead to congestive heart failure and is a common cause of patients requiring heart transplantation. In view of the progressive disease course and the acuteness of presenting symptoms, early recognition and diagnosis of the underlying etiology is essential. Genetic causes for DCM are estimated to explain 20–48% of all idiopathic patients [1]–[3]. Until now, 33 nonsyndromic DCM genes have been identified, two on the X chromosome and 31 on the autosomes, of which only one shows recessive inheritance [4]. We expect more recessive genes, since recessive forms have been shown to explain up to 16% of familial DCM [5]. Especially in young children (<10 years old) these are expected to contribute considerably to disease [6].

Protein N-glycosylation is a very common co-translational modification of many proteins, following a sequential and highly ordered pathway in the cytoplasm, endoplasmic reticulum (ER) and Golgi apparatus. Genetic defects in this pathway generally lead to a multisystem disease. These inborn errors of metabolism form the group of Congenital Disorders of Glycosylation (CDG) for which currently more than 40 different genetic defects are known [7]. Defects in the ER during the assembly of the lipid-linked oligosaccharide [8], and glycan transfer to nascent protein chains affect all N-linked proteins and typically lead to a multisystem presentation in CDG-I patients. Clinically such patients are characterized by psychomotor and intellectual disability, muscle hypotonia, seizures, ophthalmologic anomalies, failure to thrive, endocrine and coagulation abnormalities and variable dysmorphic features. Dilated cardiomyopathy in CDG is very rare and has only been described in one of the two reported families with dolichol kinase deficiency (DOLK-CDG, MIM 610768) as part of a multisystem presentation with profound muscular hypotonia, ichthyosiform skin, nystagmus, epilepsy and pulmonary infections, leading to death within the first months of life [9], and in patients with liver involvement [10] or cognitive delay [11]. On the other hand, cardiomyopathy of the hypertrophic type is common in CDG type I [12]–[14]; it is one of the lethal comorbidity factors in CDG-Ia (PMM2-CDG, MIM 212065) patients in infancy.

In this paper, we present eleven young patients (age 5–13 years) with CDG and recessive mutations in *DOLK* with a predominantly nonsyndromic presentation of DCM. In addition, we show that the main presenting symptom of DOLK-CDG is caused by deficient O-mannosylation of sarcolemmal alpha-dystroglycan.

## Results

### Clinical presentation of dilated cardiomyopathy

**Figure 1:**
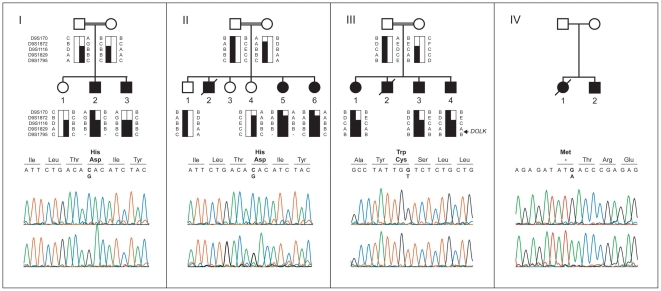
Pedigrees, haplotypes, and mutation analysis of families I through IV. Upper panels: segregation analysis of the homozygous region at 9q33.1-qter. Black bars represent haplotypes that segregate with the disease. Lower panels: chromatograms of affected family members showing the c.1222C>G, c.912G>T, and c.3G>A mutations (upper profile) and of controls (lower profile). Mutated base pairs and corresponding amino acid residues are printed in bold.

Dilated cardiomyopathy was diagnosed in several children (see pedigree; Figure 1), without significant muscular weakness or creatine kinase (CK) elevation. Central nervous system involvement, such as cerebellar ataxia, epilepsy or intellectual disability was not present in the patients, except for transient muscular hypotonia and mild developmental delay with a minor increase of CK in family IV. Decreased coagulation parameters were observed in all individuals.

### Family I

*Patient I/2*, the second male child of healthy, consanguineous parents of Druze origin was referred to the pediatric metabolic unit for evaluation of mild failure to thrive and persistent elevated transaminases during infancy. Impaired glycosylation (CDG-I) was diagnosed at the age of 10 months [15]. At 6 years of age a mild asymptomatic dilatation of the left ventricle was shown on echocardiogram. He developed acute heart failure at age 11.

*Patient I/3*, the younger brother of patient I/2, is clinically asymptomatic. At age 4 years, following the diagnosis of his brother, mildly elevated transaminases were noticed. He underwent echocardiography, which revealed mild dilated cardiomyopathy.

### Family II

*Patient II/2*, the second male child of healthy, consanguineous parents of Druze origin, was clinically healthy until the age of 9 years, when he was admitted with acute congestive heart failure and dilated cardiomyopathy of unknown etiology. He died suddenly following heart arrhythmia.

**Figure 2:**
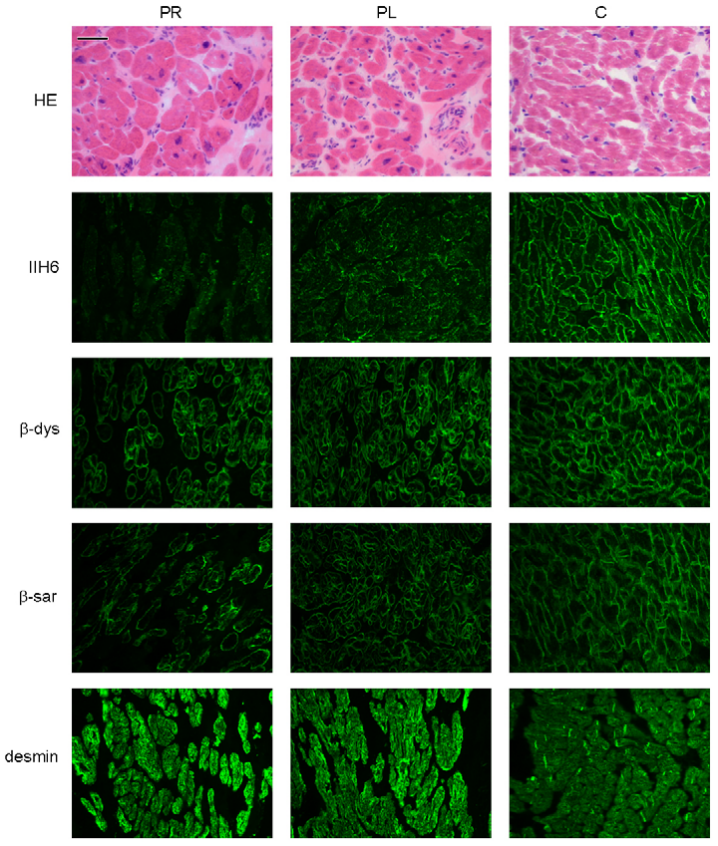
Histology and immunohistochemistry of heart sections of patient II/5 stained with haematoxylin eosin (HE), alpha dystroglycan clone IIH6C4 (IIH6), beta dystroglycan (β-dys), beta-sarcoglycan (β-sar), and desmin. PR: right ventricle of patient, PL: left ventricle of patient, C: control. Bar = 0.05 mm.

*Patient II/5*, sister of patient II/2, was diagnosed with a dilated cardiomyopathy at age 7. Biopsied ventricles of the explanted heart revealed myocyte hypertrophy and interstitial fibrosis (Figure 2), more pronounced in the left ventricle than in the right ventricle.

*Patient II/6*, a younger sister of patient II/5, had a history of mild hypotonia, failure to thrive, short stature and ichthyosiform dermatitis. She was diagnosed with mild dilated cardiomyopathy at the age of 6 years.

### Family III

*Patient III/2* was the second male child of healthy, consanguineous parents of Beduin origin. At age 9 years, he presented with progressive weakness over the last month. These symptoms led to the diagnosis of “viral myocarditis” resulting in an end-stage dilated cardiomyopathy and death after unsuccessful reanimation.

*Patient III/1*, a 13 years old sister of III/2, was found to have asymptomatic dilated cardiomyopathy following the diagnosis of her brother. Her younger brothers, patient *III/3* of 11 years old, and *III/4* of 9 years old showed asymptomatic minimal evidence of cardiomyopathy detected by repeated echocardiograms. On supportive treatment no further deterioration of the cardiac function was observed during the 3 years of follow up. Ichthyosiform dermatitis was noticed in siblings 2, 3 and 4.

### Family IV

An 11-year-old female of Indian origin with a background of learning difficulties, mild hypotonia and ichthyosis, presented with cardiac failure secondary to severe dilated cardiomyopathy. Prior to the diagnosis of CDG, her condition deteriorated; she required mechanical support and was listed for cardiac transplant. She died of thrombotic and septic complication whilst having Berlin heart as bridging procedure for transplant. Her younger brother was diagnosed with the same defect. He has mild developmental delay but cardiac function is normal.

### Glycosylation studies

**Figure 3:**
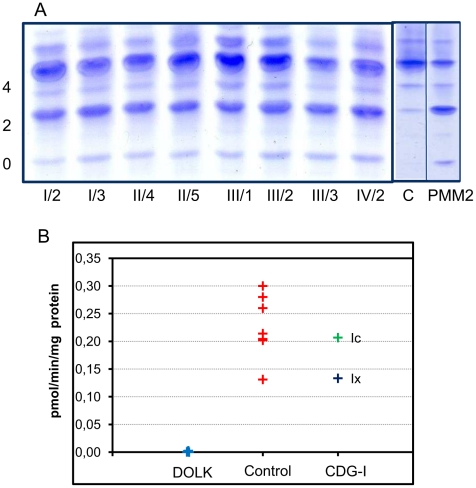
CDG biochemistry. A. N-glycosylation abnormalities in affected patients as observed by isoelectrofocusing of serum transferrin. 0, 2 and 4 indicate asialo-, disialo-, and tetrasialotransferrin isoforms. C: control, PMM2: PMM2-CDG. B. Dolichol kinase activity in patient (n = 5) and control (n = 7) fibroblasts. Microsomal fractions were incubated with γ^32^P-CTP and dolichol-19, and dolichol-^32^P production was measured.

Transferrin isoelectric focusing for analysis of N-glycosylation abnormalities was performed during metabolic screening. Convincingly abnormal profiles were found for all affected patients, showing an increase of asialo- and disialotransferrin and low or decreased tetrasialotransferrin (Figure 3). These results indicated a diagnosis of CDG type I with a genetic defect in the cytoplasm or endoplasmic reticulum. The most common subtype PMM2-CDG (CDG-Ia) was excluded by analysis of phosphomannomutase activity in patient fibroblasts. In view of the specific clinical symptoms and consanguinity in families I and II, we chose a direct homozygosity mapping approach instead of lipid-linked oligosaccharide analysis.

### Homozygosity mapping

Homozygosity mapping was performed in two Israeli families (I and II, Figure 1) using the Affymetrix GeneChip Mapping 10 K 2.0 array (family I) and the Affymetrix GeneChip Human Mapping 250 k *Nsp*I Array (family II). The largest overlapping homozygous region in families I and II was found on chromosome 9. In family I, the 19.1 Mb region at 9q33.1–9q34.3 was delimited by SNP_A-1518745 and 9pter. By using the two siblings of family II, the overlapping region could be confined to 5.0 Mb at 9q33.3 and 9q34.11, delimited by SNP_A-4223282 and SNP_A-2111464. Short tandem repeat (STR) marker analysis confirmed homozygosity of this region and showed that the haplotypes of the two Israeli families were identical (Figure 1, I and II). The three affected siblings of family III with a similar phenotype were homozygous for the same region, delimited by D9S1872 and 9qter. The overlapping homozygous region of the three families contained 117 genes. Comparison of this region with a list of candidate genes for CDG-I glycosylation defects highlighted two candidate genes known to be involved in protein N-glycosylation, *DOLPP1* and *DOLK*.

### Mutation analysis

Analysis of the protein coding sequence of *DOLK* in family I showed a homozygous missense mutation (c.1222C>G; p.His408Asp; Figure 1A). The same mutation was identified in family II. The finding in two seemingly unrelated kindreds, who reside in two different villages in Northern Israel, and the presence of an identical 5 Mb interval haplotype including the same mutation, suggest a founder event among these Druze kindreds. In family III, a homozygous c.912G>T transition was identified resulting in a p.Trp304Cys amino acid change. Both His408 and Trp304 are fully conserved down to zebrafish (Figure S1) and both SIFT [16] and PolyPhen [17] programs predict these changes to be damaging for protein function. On basis of a similar clinical presentation, *DOLK* was sequenced in DNA of family IV. A third homozygous mutation (c.3G>A, Figure 1D) was identified that removes the initiator methionine residue (p.Met1Ile), which is conserved from human to zebrafish. All three mutations were not present in >1000 healthy Caucasian controls as shown by high resolution melting analysis, by exome sequencing, and by using data from the 1000 genomes project (www.1000genomes.com) (Text S1). Analysis of the protein coding sequence of *DOLPP1* in families I and II did not show any sequence variations.

### Analysis of CTP–dependent dolichol kinase activity

Activity of dolichol kinase was assessed in patient fibroblast homogenates using dolichol-19 as acceptor and γ^32^P- cytidine 5′-triphosphate (CTP) as phosphate donor. Analysis of ^32^P incorporation into dolichol-P clearly showed strongly reduced enzyme activity for five patients of all four families investigated (Figure 3B). Fibroblasts from CDG-I patients with a different genetic defect showed dolichol kinase activity comparable to controls.

### Functional analysis of *DOLK* mutant alleles in the temperature sensitive yeast *sec59* mutant

**Figure 4:**
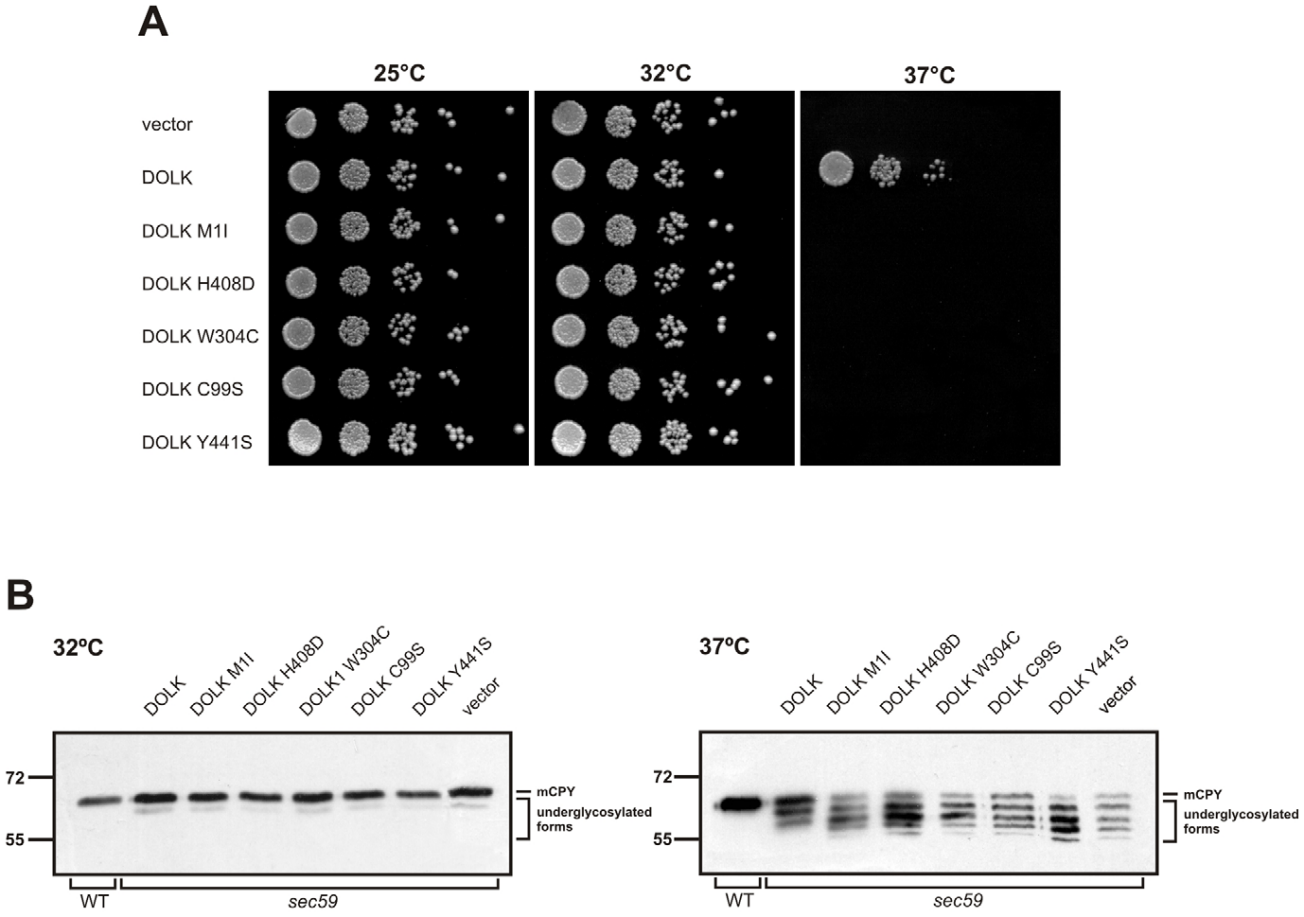
Functional analysis of *DOLK* mutant alleles in the temperature sensitive yeast *sec59* mutant. A. Serial dilutions of *sec59* mutant, transformed with plasmids, harboring either wild-type *DOLK*, mutant alleles, or empty vector, were plated on YPD media and incubated at 25, 32, or 37°C. B. Glycosylation pattern of CPY in *sec59* mutant transformed with wild-type and mutant *DOLK* alleles. Cells were cultivated at the permissive temperature of 25°C and shifted for 10 h to 32 or 37°C, followed by immunoblotting as described [19]. The positions of mature CPY (mCPY) and underglycosylated forms lacking one to four N-glycans are indicated.

*SEC59* is the yeast ortholog of *DOLK* [18]. A *sec59* yeast mutant that displays temperature sensitive lethality as well as an underglycosylation of glycoproteins at the restrictive temperature was used to further confirm the non-functionality of the mutations in our patients. In addition, we have compared the novel mutations with the previously reported mutations in the two DOLK-CDG patients. All strains showed comparable growth at the permissive temperatures of 25 or 32°C, whereas at the restrictive temperature of 37°C only wild-type *DOLK* supported growth (Figure 4A). N-glycosylation of the same mutant alleles was assessed by western blotting of the vacuolar glycoprotein carboxypeptidase Y (CPY), containing four N-glycan chains (Figure 4B). At the restrictive temperature CPY is underglycosylated in *sec59* cells, visualized by the appearance of glycoforms lacking one to four N-glycan chains. Consistent with the cell growth results, wild-type *DOLK* was able to restore the glycosylation of CPY at 37°C, as evidenced by a shift to the more mature forms of CPY and a decrease of underglycosylated isoforms. All mutants failed to restore glycosylation to the same extent as wild-type *DOLK*. The p.Tyr441Ser and p.Cys99Ser mutants showed no or marginal improvement, respectively, as compared to *sec59* cells. The three new mutants, however, improved the glycosylation to a higher extent as the glycosylation patterns showed a more prominent band of CPY with two N-glycans as compared to CPY with only one N-glycan.

### Glycosylation studies in heart biopsy and *DOLK* expression

**Figure 5:**
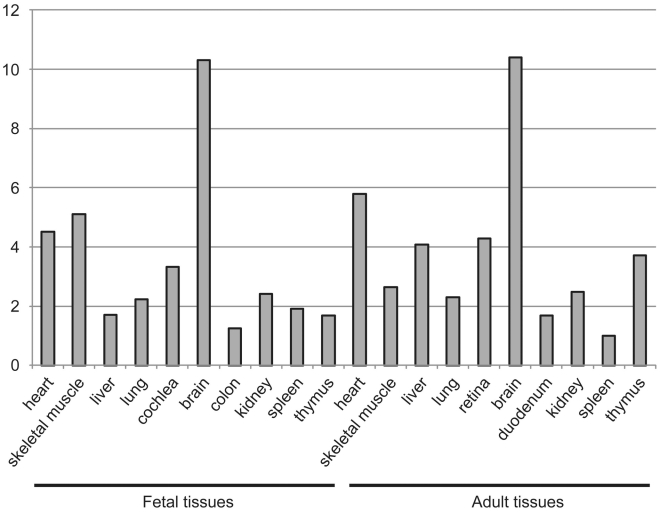
Expression of dolichol kinase by mRNA expression analysis in human fetal and adult tissues. Relative expression levels are given as the fold change in comparison to the tissue with the lowest expression level.

To explain the tissue-restricted clinical phenotype in our patient group, we performed expression analysis of *DOLK* in fetal and adult tissue and biochemical analysis of the dolichol-phosphate dependent N-glycosylation and O-mannosylation. Highest expression levels of *DOLK* mRNA were found in fetal and adult brain, followed by skeletal muscle and heart in fetal tissue and heart in adult (Figure 5).

**Figure 6:**
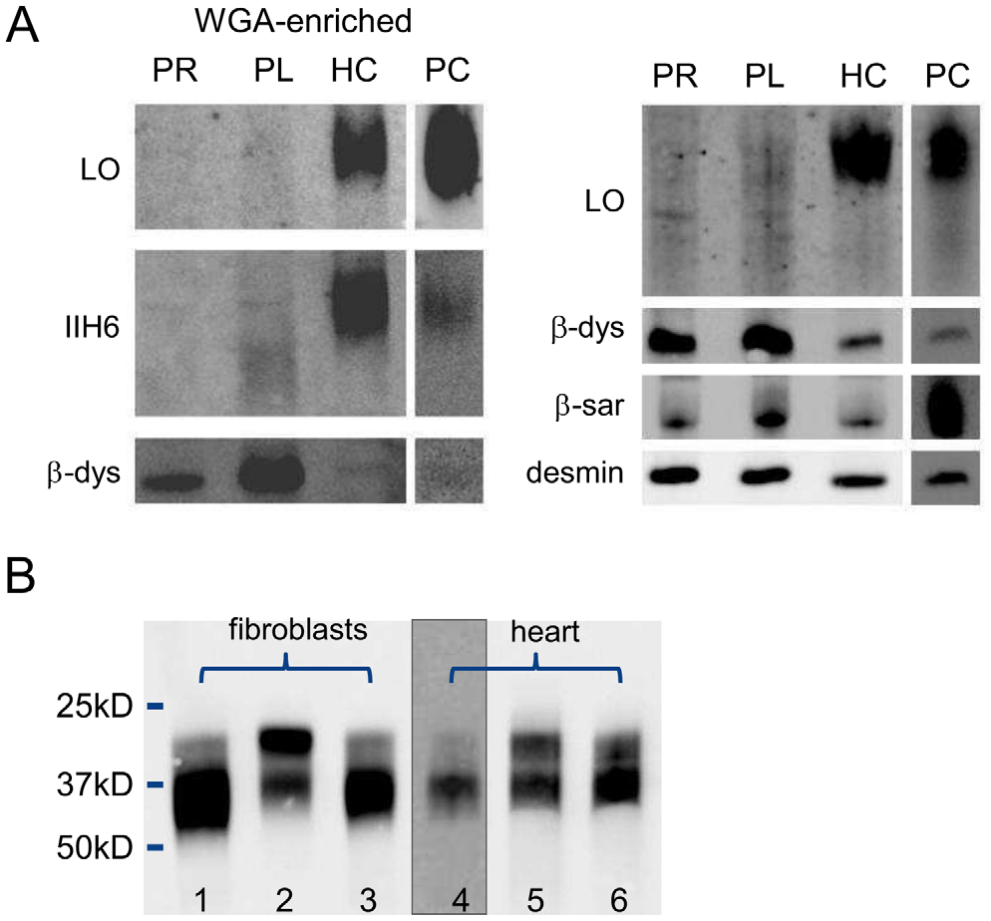
Western blotting for analysis of O-mannosylation and N-glycosylation. O-mannosylation (A) and N-glycosylation (B). A. Heart muscle homogenates were used with (left panel) or without (right panel) WGA-enrichment and stained for desmin, β-dystroglycan, β-sarcoglyan, glycosylated α-dystroglycan (IIH6) and used for functional analysis of laminin binding. PR: patient II/5 right ventricle; PL: patient II/5 left ventricle; HC: healthy control; PC: pathological cardiomyopathy control. B. Patient and control fibroblasts and heart tissue homogenates were used for western blotting of CD63. Lanes 1 and 4: control; lane 2: DPM1-CDG; lane 3: DOLK-CDG; lane 5: patient right ventricle; lane 6: patient left ventricle.

Dolichol-P is required for N-glycosylation in the ER. In addition, dolichol-P is converted to dolichol-P-mannose, the monosaccharide donor for N-glycosylation inside the ER lumen and for O-mannosylation of alpha-dystroglycan. O-mannosylation was assessed by direct immunofluorescence staining of a frozen heart biopsy with the IIH6 antibody directed against the O-mannosyl glycans of alpha-dystroglycan. Reduced and/or fragmented staining was observed, more pronounced in the right ventricle. The intensity of the sarcolemmal proteins beta-dystroglycan and beta-sarcoglycan was normal, while the intensity of intracellular desmin was somewhat increased (Figure 2). Western blotting was performed on heart muscle homogenates. IIH6 staining of WGA-enriched fractions was reduced (Figure 6A), which was confirmed in the laminin-overlay (LO) assay showing a reduction of the laminin-binding capacity of alpha-dystroglycan. To correct for muscle specific staining, western blotting was performed on non-enriched heart homogenates (Figure 6A) using anti-desmin and anti-β-sarcoglycan primary antibodies. Equal signals were observed for control and patient materials. However, the laminin-overlay assay clearly showed a reduction in signal intensity, similar to the results in WGA-enriched fractions. Additional control studies were performed in heart tissues of patients with idiopathic cardiomyopathy (Figure 6A, PC), with similar results as for the healthy controls (HC). N-glycosylation was analyzed by western blotting of the lysosomal glycoprotein CD63 (LAMP3). In fibroblasts of a DPM1-CDG patient, a clear shift was seen to a lower glycosylated CD63 isoform, indicating aberrant N-glycosylation of CD63 as compared to control fibroblasts. Glycosylation of CD63 in dolichol kinase deficient fibroblasts was comparable to controls (Figure 6B). Analysis of homogenized heart tissue showed a shift in CD63 isoforms in dolichol kinase deficient heart material as compared to control heart, indicating reduced N-glycosylation.

## Discussion

In a cohort of 11 patients, presenting primarily with nonsyndromic dilated cardiomyopathy at the age of 5–13 years, we identified three separate mutations in *DOLK* as the underlying cause of disease. Some of the patients showed mild additional clinical symptoms, such as ichthyosis, failure to thrive and mild neurological involvement. In contrast, the two families with *DOLK* mutations originally described by Kranz et al [9] showed a severe congenital multisystem phenotype including a variable presentation of cardiac failure, severe muscular hypotonia, and ichthyosis, with epilepsy due to hypsarrhythmia, microcephaly and visual impairment, leading to death within 6 months after birth. Dolichol kinase is an endoplasmic reticulum resident protein with a cytidine-5′-triphosphate (CTP) binding pocket in the C-terminal domain that is exposed to the cytoplasmic face [20]. The exact catalytic mechanism, the hydrophobic binding sites for dolichol, and a possible role in dolichol-P recycling have not been clarified as yet. Our and the previously identified mutations occur in or near transmembrane domains, not associated with a specific function (Figure S1). Functional investigation of these mutant alleles in the temperature sensitive yeast strain *sec59*, deficient in dolichol kinase activity, showed sustained reduced growth at 37°C. Moreover, a less severe underglycosylation of CPY was found in the three new mutants as compared to the two mutations from the previous report, which is in agreement with the milder clinical phenotype in our families.

*DOLK* mutations result in abnormal N-glycosylation as determined by analysis of serum transferrin glycosylation in our patients. Remarkably, only minor classical symptoms of CDG-I could be identified in the patients described here, such as increased liver transaminases in some and a slight decrease in coagulation parameters in all patients. No signs of cerebellar hypoplasia were observed, as commonly seen in the most frequent CDG subtype PMM2-CDG. On the other hand, dilated cardiomyopathy is uncommon in CDG patients with an N-glycosylation defect. A single case out of more than 40 known ALG6-CDG (MIM 603147) patients was reported with a multisystem presentation including DCM [21]. In DPM3-CDG (MIM 612937), DCM was reported as minor symptom compared to the muscular dystrophy [22]. As deduced from deficient IIH6 staining in skeletal muscle, both clinical symptoms were linked to deficient O-mannosylation of alpha-dystroglycan. In the disorders of dystroglycan O-mannosylation, a subgroup of the congenital muscular dystrophies, DCM is commonly observed in combination with limb-girdle muscular dystrophy at the milder end of the spectrum. Patients with dilated cardiomyopathy and no or minimal muscle involvement were reported with mutations in fukutin (*FKTN*, [23]) and fukutin-related protein (*FKRP*, [24]), showing reduced laminin binding capacity of alpha-dystroglycan in heart muscle biopsies. The involvement of dystroglycan O-mannosylation in the phenotype of our cohort of dolichol kinase deficient patients was shown by reduced IIH6 staining in frozen heart biopsy material. Western blot analysis showed a reduction in the laminin binding capacity of alpha-dystroglycan, thereby confirming a loss of alpha-dystroglycan function. Recently, the loss of functional alpha-dystroglycan as extracellular receptor in cardiac myocytes was shown to be the cause of dilated cardiomyopathy in mutant mice [25]. Dystroglycan was postulated as an important extracellular matrix receptor to limit the damage of cardiomyocyte membranes after exercise-induced stress to individual cells.

**Figure 7:**
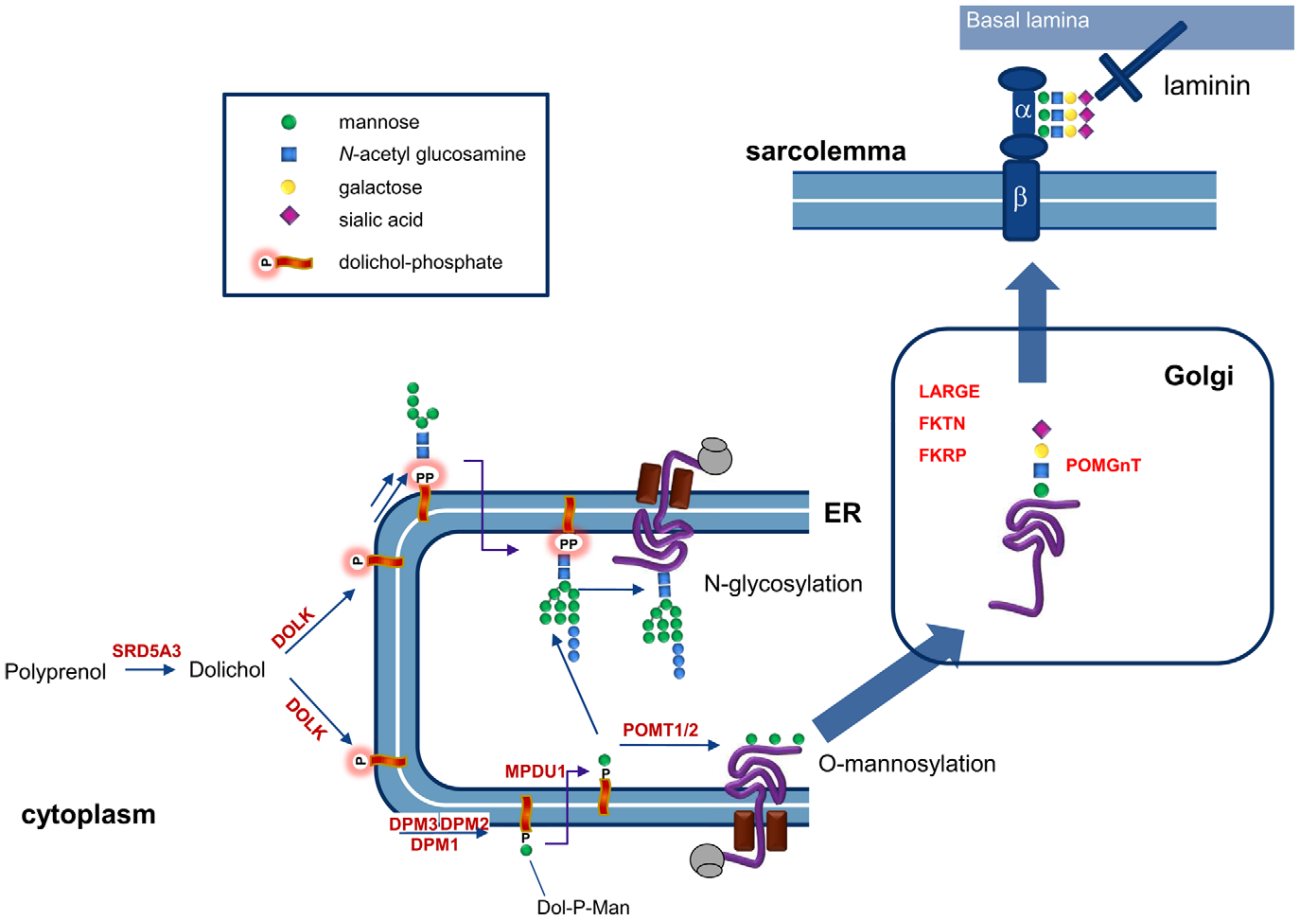
Involvement of dolichol kinase (DOLK) in N-glycosylation and O-mannosylation in the ER. The enzymes involved in O-mannosylation of alpha-dystroglycan are indicated. Dystroglycan is proteolytically cleaved into a beta and alpha subunit. The mucin domain of α-DG contains the O-mannosyl glycans that are required for binding to the basal lamina via laminin. In addition, mucin O-glycans and phosphorylated O-mannosyl glycans (not shown) are present.

O-Mannosylation of alpha-dystroglycan (Figure 7) involves the protein O-mannosyltransferases POMT1 and POMT2 and the GlcNAc transferase POMGnT1. In addition fukutin, fukutin-related protein and LARGE have been shown to be involved in O-mannosylation, where LARGE is involved in a phosphorylation process of the O-mannosyl glycan [26]. Defects in these six genes have been described as cause for the dystroglycanopathies [27], explaining disease in only about 50% of the patients [28]. Defects in the biosynthetic genes of the sugar donor dolichol-P-mannose required for the O-mannosylation process, like DPM3 [22] and likely DPM1 [29], [30], result in abnormal dystroglycan O-mannosylation. Here, we show that mutations in dolichol kinase also lead to reduced dystroglycan O-mannosylation, likely via reduced availability of dolichol-P-mannose. This is supported by previous studies in yeast cells [31]: amphomycin, which binds to and inhibits the use of dolichol-phosphate, was shown to reduce the production of dolichol-P-mannose with a subsequent reduction of protein O-mannosylation. Interestingly, N-glycosylation of the O-mannosylating enzymes POMT1 and POMT2 was shown to be required for their activity [32]. This implies that in dolichol kinase deficiency, O-mannosylation could be reduced via two independent mechanisms, i.e. via reduced availability of dolichol-P-mannose and via reduced activity of O-mannosylation enzymes due to deficient N-glycosylation. Possibly, this leads to increased susceptibility of the O-mannosylation pathway in defects of dolichol-P or dolichol-P-mannose synthesis. In contrast, the clinical phenotype of CDG-If (MPDU1-CDG, MIM 609180) does not include muscular dystrophy or dilated cardiomyopathy in spite of the reduced availability of dolichol-P-mannose in the ER lumen in this disease [33], [34]. Also, the recently described polyprenol reductase SRD5A3-CDG (MIM 612379) does not show signs of a congenital muscular dystrophy [35], [36]. Apparently, additional factors play a role in determining the clinical outcome in deficiencies of dolichol-P-mannose synthesis or utilization. For SRD5A3, a by-pass synthesis route for dolichol was postulated [35], while both dolichol and dolichol-phosphate could have a function on their own in organelle membrane fluidity [37]. Clearly, many factors in dolichol and dolichol-phosphate homeostasis remain to be discovered [38], which could differentially affect the clinical outcome in dolichol cycle defects.

In conclusion, we have shown that dolichol kinase deficiency results in abnormal N-glycosylation and reduced O-mannosylation of alpha-dystroglycan, leading to a clinical phenotype of dilated cardiomyopathy. This new entity of cardiomyopathy warrants screening for glycosylation defects in any patient with idiopathic DCM. Dolichol kinase deficiency may initially present with mild or asymptomatic DCM which may deteriorate, underlining the necessity to follow these young patients closely.

## Methods

### Patient description

Three families residing in the Galilee regions of Northern Israel and one Indian family were clinically and genetically investigated. Over the past five years, 11 children have been diagnosed as suffering from an autosomal recessive dilated cardiomyopathy associated with CDG type I transferrin isoelectric focusing profiles in serum (see pedigrees in Figure 1). Nine patients and 11 of their close relatives were included in the study. The study protocol was approved by the Institutional Ethics Review Committee and by the National Committee for Genetic Studies of the Israeli Ministry of Health. Informed consent was obtained from all participants and their legal guardians.

### CDG diagnostics

Transferrin isoelectric focusing was carried out as described before [39]. The clinical symptoms did not show any indication for the presence of fructosemia or galactosemia as possible secondary cause for CDG type I transferrin isoelectric focusing profiles. A protein polymorphism was excluded by neuraminidase digestion of the samples and by the normal profiles of both parents. Phosphomannomutase activity was measured in patient fibroblasts according to [40]. For analysis of dolichol kinase activity, fibroblast homogenates were incubated with [γ-^32^P]cytidine 5′-triphosphate and dolichol-19 and the formation of ^32^P-dolichol was measured according to [9] and described in detail in Text S1.

### Homozygosity mapping

Genomic DNA was extracted from peripheral blood lymphocytes using standard salting out procedures [41]. Genotyping was performed using the Affymetrix *Nsp*I 250 K SNP array. All SNP array experiments were performed and analyzed according to manufacturer's protocols (Affymetrix, Santa Clara, CA, USA). Homozygosity mapping was performed using PLINK v1.06 [42], using a homozygous window of 50 SNPs tolerating two heterozygous SNPs and ten missing SNPs per window.

### Mutation analysis

Primer sequences for amplification of the only exon of *DOLK* (GenBank ID NM_014908.3) are shown in Table S1. PCR products were sequenced using the ABI PRISM BigDye Terminator Cycle Sequencing V2.0 Ready Reaction Kit and analyzed with the ABI PRISM 3730 DNA analyzer (Applied Biosystems, Foster City, USA).

### Immunohistochemistry and western blotting

Immunohistochemistry was performed by incubation of heart tissue sections with monoclonal antibodies against alpha-dystroglycan, beta-dystroglycan, beta-sarcoglycan or desmin (Text S1).

WGA-enriched and non-enriched heart homogenates were used for western blotting of CD63, beta-dystroglycan, desmin, beta-sarcoglycan and alpha-dystroglycan and for the laminin overlay assay as described ([43] and Text S1).

### Expression in yeast

For expression of *DOLK*, the following strain was used: *MAT*a *sec59 ura3-52*. Cells were grown in selective YNB (yeast nitrogen base) medium (0.67% YNB, 0.5% casamino acids and 2% glucose) or in YPD medium (1% yeast extract, 2% bacto-peptone and 2%glucose). For growth on plates 2% agar was added. To construct the yeast expression plasmids, the *DOLK* open reading frame (encoded by a single exon) was PCR amplified (Phusion High-Fidelity DNA Polymerase, New England Biolabs) from the chromosomal DNA of patients I-3, III-1, and IV-2 and a healthy control with primers engineered with *Hind*III and *Bam*HI restriction sites at the 5′and 3′ ends, respectively (Table S1). For patient IV-2, where the mutation is located within the primer region, the mutation was introduced in the primer. All four products were subcloned into the pCR4-TOPO vector (Invitrogen, Breda, The Netherlands). The two mutations described previously by Kranz et al. [9] were introduced in the WT-*DOLK* containing plasmid by site-directed mutagenesis. Subsequently, the WT and mutated forms of *DOLK* were digested with *Hind*III/*Bam*H1 and ligated into the *Hind*III/*Bam*HI digested vector pVT100-ZZ, thereby placing *DOLK* under the control of the constitutive *ADH1* (alcohol dehydrogenase 1) promoter. The correct sequences were verified by sequencing the entire coding region of the constructs. Transformation into yeast cells was carried out using standard techniques [19].

## Supporting Information

Figure S1 Amino acid changes. A) Schematic representation of DOLK including the positions of all mutations identified thus far (this paper and [9]). Transmembrane domains according to Haeuptle [10] are represented by the numbered black boxes. B) Alignment of transmembrane domains 9 and 12 from human (Homo sapiens), mouse (Mus musculus), chicken (Gallus gallus) and zebrafish (Danio rerio). The substituted amino acid residues p.Trp304 and p.His408 are highlighted by the black boxes and conserved in all four species.
(JPG)

Table S1 Primer sequences of primers used for direct DNA sequencing, HRM, and QPCR analysis of *DOLK* (NM_014908.3). In addition, primers for short tandem repeat (STR) marker analysis, and for cloning and site-directed mutagenesis of *DOLK* are shown. STR primers are given without their M13 tails. The restriction sites used for cloning are underlined in the respective primers. Introduced nucleotide mutations are printed in bold.
(DOC)

Text S1 Details on biochemical and genetic methods.
(DOCX)

## References

[1] Michels VV, Moll PP, Miller FA, Tajik AJ, Chu JS (1992). The frequency of familial dilated cardiomyopathy in a series of patients with idiopathic dilated cardiomyopathy.. N Engl J Med.

[2] Grunig E, Tasman JA, Kucherer H, Franz W, Kubler W (1998). Frequency and phenotypes of familial dilated cardiomyopathy.. J Am Coll Cardiol.

[3] Baig MK, Goldman JH, Caforio AL, Coonar AS, Keeling PJ (1998). Familial dilated cardiomyopathy: cardiac abnormalities are common in asymptomatic relatives and may represent early disease.. J Am Coll Cardiol.

[4] Hershberger RE, Siegfried JD (2011). Update 2011: clinical and genetic issues in familial dilated cardiomyopathy.. J Am Coll Cardiol.

[5] Sinagra G, Di Lenarda A, Brodsky GL, Taylor MR, Muntoni F (2001). Current perspective new insights into the molecular basis of familial dilated cardiomyopathy.. Ital Heart J.

[6] Seliem MA, Mansara KB, Palileo M, Ye X, Zhang Z (2000). Evidence for autosomal recessive inheritance of infantile dilated cardiomyopathy: studies from the Eastern Province of Saudi Arabia.. Pediatr Res.

[7] Jaeken J (2011). Congenital disorders of glycosylation (CDG): it's (nearly) all in it!. J Inherit Metab Dis.

[8] Haeuptle MA, Hennet T (2009). Congenital disorders of glycosylation: an update on defects affecting the biosynthesis of dolichol-linked oligosaccharides.. Hum Mutat.

[9] Kranz C, Jungeblut C, Denecke J, Erlekotte A, Sohlbach C (2007). A defect in dolichol phosphate biosynthesis causes a new inherited disorder with death in early infancy.. Am J Hum Genet.

[10] Gehrmann J, Sohlbach K, Linnebank M, Bohles HJ, Buderus S (2003). Cardiomyopathy in congenital disorders of glycosylation.. Cardiol Young.

[11] Footitt EJ, Karimova A, Burch M, Yayeh T, Dupre T (2009). Cardiomyopathy in the congenital disorders of glycosylation (CDG): a case of late presentation and literature review.. J Inherit Metab Dis.

[12] Marquardt T, Hulskamp G, Gehrmann J, Debus V, Harms E (2002). Severe transient myocardial ischaemia caused by hypertrophic cardiomyopathy in a patient with congenital disorder of glycosylation type Ia.. Eur J Pediatr.

[13] Noelle V, Knuepfer M, Pulzer F, Schuster V, Siekmeyer W (2005). Unusual presentation of congenital disorder of glycosylation type 1a: congenital persistent thrombocytopenia, hypertrophic cardiomyopathy and hydrops-like aspect due to marked peripheral oedema.. Eur J Pediatr.

[14] van de Kamp JM, Lefeber DJ, Ruijter GJ, Steggerda SJ, den Hollander NS (2007). Congenital disorder of glycosylation type Ia presenting with hydrops fetalis.. J Med Genet.

[15] Iancu TC, Mahajnah M, Manov I, Cherurg S, Knopf C (2007). The liver in congenital disorders of glycosylation: ultrastructural features.. Ultrastruct Pathol.

[16] Ng PC, Henikoff S (2002). Accounting for human polymorphisms predicted to affect protein function.. Genome Res.

[17] Ramensky V, Bork P, Sunyaev S (2002). Human non-synonymous SNPs: server and survey.. Nucleic Acids Res.

[18] Fernandez F, Shridas P, Jiang S, Aebi M, Waechter CJ (2002). Expression and characterization of a human cDNA that complements the temperature-sensitive defect in dolichol kinase activity in the yeast *sec59-1* mutant: the enzymatic phosphorylation of dolichol and diacylglycerol are catalyzed by separate CTP-mediated kinase activities in *Saccharomyces cerevisiae*.. Glycobiology.

[19] Absmanner B, Schmeiser V, Kämpf M, Lehle L (2010). Biochemical characterization, membrane association and identification of amino acids essential for the function of Alg11 from *Saccharomyces cerevisiae*, an alpha1,2-mannosyltransferase catalysing two sequential glycosylation steps in the formation of the lipid-linked core oligosaccharide.. Biochem J.

[20] Shridas P, Waechter CJ (2006). Human dolichol kinase, a polytopic endoplasmic reticulum membrane protein with a cytoplasmically oriented CTP-binding site.. J Biol Chem.

[21] Al-Owain M, Mohamed S, Kaya N, Zagal A, Matthijs G (2010). A novel mutation and first report of dilated cardiomyopathy in ALG6-CDG (CDG-Ic): a case report.. Orphanet J Rare Dis.

[22] Lefeber DJ, Schönberger J, Morava E, Guillard M, Huyben KM (2009). Deficiency of Dol-P-Man synthase subunit DPM3 bridges the congenital disorders of glycosylation with the dystroglycanopathies.. Am J Hum Genet.

[23] Murakami T, Hayashi YK, Noguchi S, Ogawa M, Nonaka I (2006). Fukutin gene mutations cause dilated cardiomyopathy with minimal muscle weakness.. Ann Neurol.

[24] Boito CA, Melacini P, Vianello A, Prandini P, Gavassini BF (2005). Clinical and molecular characterization of patients with limb-girdle muscular dystrophy type 2I.. Arch Neurol.

[25] Michele DE, Kabaeva Z, Davis SL, Weiss RM, Campbell KP (2009). Dystroglycan matrix receptor function in cardiac myocytes is important for limiting activity-induced myocardial damage.. Circ Res.

[26] Yoshida-Moriguchi T, Yu L, Stalnaker SH, Davis S, Kunz S (2010). O-mannosyl phosphorylation of alpha-dystroglycan is required for laminin binding.. Science.

[27] Barresi R, Campbell KP (2006). Dystroglycan: from biosynthesis to pathogenesis of human disease.. J Cell Sci.

[28] van Reeuwijk J, Brunner H, van Bokhoven H (2005). Glyc-O-genetics of Walker-Warburg syndrome.. Clin Genet.

[29] Kim S, Westphal V, Srikrishna G, Mehta DP, Peterson S (2000). Dolichol phosphate mannose synthase (DPM1) mutations define congenital disorder of glycosylation Ie (CDG-Ie).. J Clin Invest.

[30] Imbach T, Schenk B, Schollen E, Burda P, Stutz A (2000). Deficiency of dolichol-phosphate-mannose synthase-1 causes congenital disorder of glycosylation type Ie.. J Clin Invest.

[31] Rroyo-Flores BL, Calvo-Mendez C, Flores-Carreon A, Lopez-Romero E (1995). Biosynthesis of glycoproteins in Candida albicans: activity of dolichol phosphate mannose synthase and protein mannosylation in a mixed membrane fraction.. Microbiology.

[32] Manya H, kasaka-Manya K, Nakajima A, Kawakita M, Endo T (2010). Role of N-glycans in maintaining the activity of protein O-mannosyltransferases POMT1 and POMT2.. J Biochem.

[33] Kranz C, Denecke J, Lehrman MA, Ray S, Kienz P (2001). A mutation in the human MPDU1 gene causes congenital disorder of glycosylation type If (CDG-If).. J Clin Invest.

[34] Schenk B, Imbach T, Frank CG, Grubenmann CE, Raymond GV (2001). MPDU1 mutations underlie a novel human congenital disorder of glycosylation, designated type If.. J Clin Invest.

[35] Cantagrel V, Lefeber DJ, Ng BG, Guan Z, Silhavy JL (2010). SRD5A3 is required for converting polyprenol to dolichol and is mutated in a congenital glycosylation disorder.. Cell.

[36] Morava E, Wevers RA, Cantagrel V, Hoefsloot LH, Al-Gazali L (2010). A novel cerebello-ocular syndrome with abnormal glycosylation due to abnormalities in dolichol metabolism.. Brain.

[37] Valtersson C, van Duijn G, Verkleij AJ, Chojnacki T, de Kruijff B (1985). The influence of dolichol, dolichol esters, and dolichyl phosphate on phospholipid polymorphism and fluidity in model membranes.. J Biol Chem.

[38] Cantagrel V, Lefeber DJ (2011). From glycosylation disorders to dolichol biosynthesis defects: a new class of metabolic diseases.. J Inherit Metab Dis.

[39] de Jong JG, van Noort WL, van Eijk HG (1994). Optimized separation and quantitation of serum and cerebrospinal fluid transferrin subfractions defined by differences in iron saturation or glycan composition.. Adv Exp Med Biol.

[40] Pirard M, Matthijs G, Heykants L, Schollen E, Grunewald S (1999). Effect of mutations found in carbohydrate-deficient glycoprotein syndrome type IA on the activity of phosphomannomutase 2.. FEBS Lett.

[41] Miller SA, Dykes DD, Polesky HF (1988). A simple salting out procedure for extracting DNA from human nucleated cells.. Nucleic Acids Res.

[42] Purcell S, Neale B, Todd-Brown K, Thomas L, Ferreira MA, Bender D (2007). PLINK: a tool set for whole-genome association and population-based linkage analyses.. Am J Hum Genet.

[43] Michele DE, Barresi R, Kanagawa M, Saito F, Cohn RD (2002). Post-translational disruption of dystroglycan-ligand interactions in congenital muscular dystrophies.. Nature.

