# Supplementary figures and images for: Autosomal Recessive Dilated Cardiomyopathy due to DOLK Mutations Results from Abnormal Dystroglycan O-Mannosylation
Source: PLoS Genet. 2011 Dec 29;7(12):e1002427. doi: 10.1371/journal.pgen.1002427 (PMC3248466; doi:10.1371/journal.pgen.1002427)

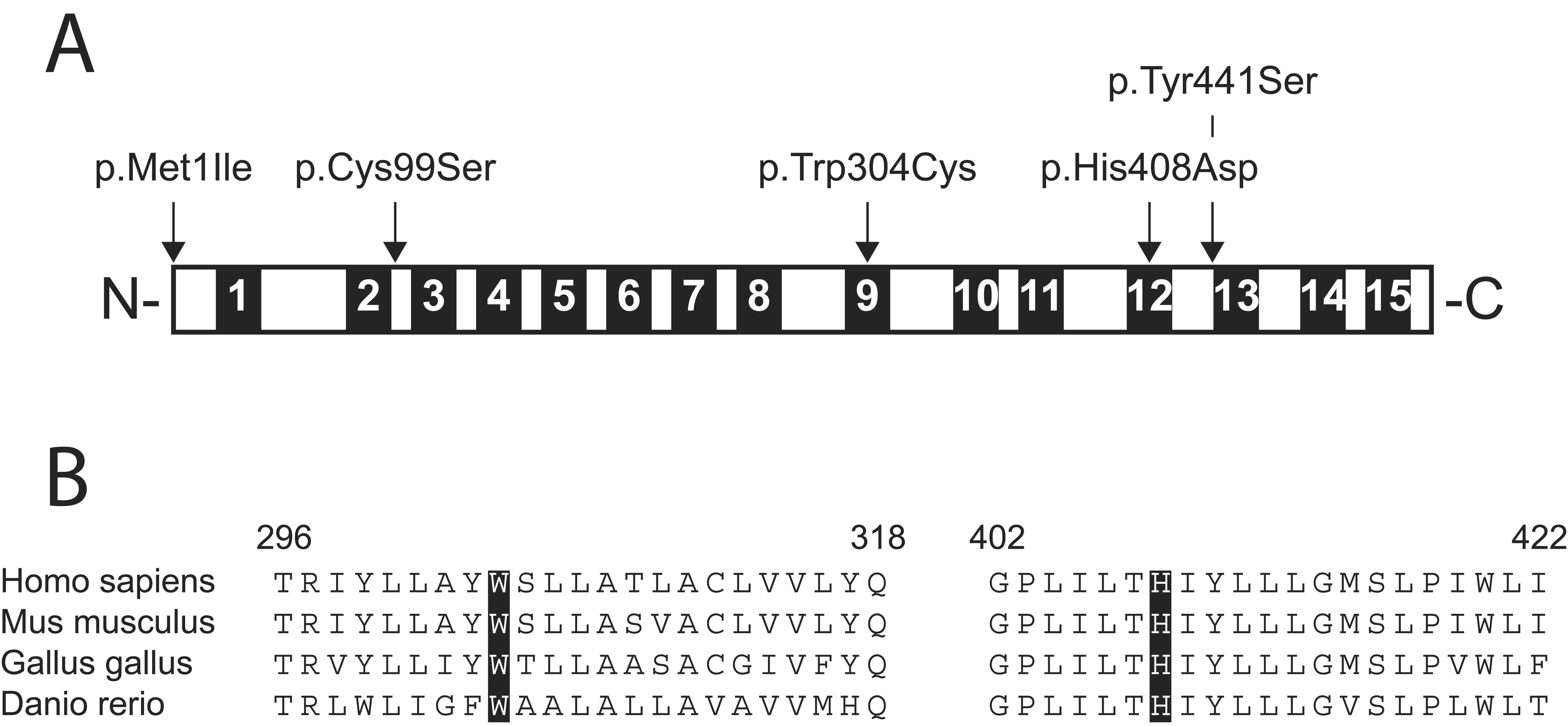

Supplement: Figure S1 — Amino acid changes. A) Schematic representation of DOLK including the positions of all mutations identified thus far (this paper and [9]). Transmembrane domains according to Haeuptle [10] are represented by the numbered black boxes. B) Alignment of transmembrane domains 9 and 12 from human (Homo sapiens), mouse (Mus musculus), chicken (Gallus gallus) and zebrafish (Danio rerio). The substituted amino acid residues p.Trp304 and p.His408 are highlighted by the black boxes and conserved in all four species. (JPG) [file pgen.1002427.s001.jpg]
