# Supplementary material for: Autosomal Recessive Dilated Cardiomyopathy due to DOLK Mutations Results from Abnormal Dystroglycan O-Mannosylation
Source: PLoS Genet. 2011 Dec 29;7(12):e1002427. doi: 10.1371/journal.pgen.1002427 (PMC3248466; doi:10.1371/journal.pgen.1002427)
Supplement: Table S1 — Primer sequences of primers used for direct DNA sequencing, HRM, and QPCR analysis of DOLK (NM_014908.3). In addition, primers for short tandem repeat (STR) marker analysis, and for cloning and site-directed mutagenesis of DOLK are shown. STR primers are given without their M13 tails. The restriction sites used for cloning are underlined in the respective primers. Introduced nucleotide mutations are printed in bold. (DOC) [file pgen.1002427.s002.doc]

Table S1

|  | Forward primer (5’→3’) | Reverse primer (5’→3’) |
| --- | --- | --- |
|  | Sequencing | |
| Exon 1_1 | acatcccgcctccaattc | aaacactactgccgcctctg |
| Exon 1_2 | gtctagggcgtccgtagtcg | tccaggacttcgatcacctc |
| Exon 1_3 | cagtgccaaccaacacttg | gtgcttcttggactcggaag |
| Exon 1_4 | cttggtgtggtcctaccctg | catggtgctaccgaagatgg |
| Exon 1_5 | atcaagcctttgggtcacac | ttaaatatcagcaaggctggg |
|  |  |  |
|  | HRM analysis | |
| c.3G>A | aagtgcctccctctgcttg | aaacactactgccgcctctg |
|  |  |  |
|  | QPCR expression analysis | |
| *GUSB* (NM_000181.1) | agagtggtgctgaggattgg | ccctcatgctctagcgtgtc |
| *PPIB* (NM_000942.4) | cggaaagactgttccaaaaac | gattacacgatggaatttgctg |
| *DOLK* (NM_014908.3) | acctcatgacctgtgtgctg | tagatgcgggtgtctgtctg |
|  |  |  |
|  | STR markers analysis | |
| D9S170 | cctgaattcctcatctgaaagc | tctaattatgcagactccgtgtg |
| D9S1872 | catagaaactggggcaggag | atgctggagttgtgggaatc |
| D9S1116 | tttctacccagtagtttcattcc | accggacatgtgtgtaagtg |
| D9S1829 | gctgagtggaagaagcgaac | atgcagcaagaccccatc |
| D9S1795 | tagcacagtgaaagcccatc | tggccattgatttatttaggg |
|  |  |  |
|  | Cloning | |
| *DOLK* (NM_014908.3) | cccaagcttaaaatgacccgagagtgcccatctccggccccg | cgggatccggccatcagcaatatcaggaggtag |
| *DOLK* (p.Met1Ile) | cccaagcttaaaat**a**acccgagagtgcccatctccggccccg | cgggatccggccatcagcaatatcaggaggtag |
|  |  |  |
|  | Site-directed mutagenesis | |
| *DOLK* (p.Cys99Ser) | catgaaggagcgg**a**gccagactgctgggaaccc | ggttcccagcagtctggc**t**ccgctccttcatg |
| *DOLK* (p.Tyr441Ser) | gggccctcgtcccct**c**tgccggtgtcctggctgtggg | cccacagccaggacaccggca**g**aggggacgagggccc |
|  |  |  |
