# Supplementary material for: Autosomal Recessive Dilated Cardiomyopathy due to DOLK Mutations Results from Abnormal Dystroglycan O-Mannosylation
Source: PLoS Genet. 2011 Dec 29;7(12):e1002427. doi: 10.1371/journal.pgen.1002427 (PMC3248466; doi:10.1371/journal.pgen.1002427)
Supplement: Text S1 — Details on biochemical and genetic methods. (DOCX) [file pgen.1002427.s003.docx]

*Supplementary data*

**Recessive Dilated Cardiomyopathy due to DOLK Mutations Results from Abnormal Dystroglycan O-Mannosylation**

D.J. Lefeber et al. Department of Neurology, Institute for Genetic and Metabolic Disease, Radboud University Nijmegen Medical Centre, Geert Grooteplein 10, 6525 GA Nijmegen, The Netherlands. Phone: +31 24 3614428; Fax: +31 24 3618900. E-mail: [D.Lefeber@neuro.umcn.nl](mailto:D.Lefeber@neuro.umcn.nl)

**Short tandem repeat (STR) marker analysis**

Primers to amplify polymorphic short tandem repeat markers on 9q34.11 were designed by using the Primer3 program (<http://frodo.wi.mit.edu/primer3/>) [1]. An M13 tail was added to the 5’ and 3’-ends of the primers. Markers were amplified by using an M13 forward primer labeled with one of the fluorophores, FAM, VIC, NED and ROX, at the 5’-end [2] and a M13 reverse primer with a 5’-gtttctt-3’ added to its 5’-end to reduce tailing [3]. Primer sequences are shown in Table S1. Final PCR products were mixed with eight volumes of formamide and half a volume of Genescan^TM^ 500(-250) LIZ size standard (Applied Biosystems, Foster City, USA), and analysed with the ABI PRISM 3730 DNA analyzer (Applied Biosystems, Foster City, USA). The results were evaluated by Genemapper (Applied Biosystems, Foster City, USA).

**STR marker and PCR amplification conditions**

In case of STR marker and *DOLK* mutation analysis the PCR-Mix consisted of 1x PCR buffer (Invitrogen, Leek, The Netherlands), 3 mM MgCl_2_, 250 µM dNTP’s (Invitrogen), 2 U Taq polymerase (Invitrogen), 0.3 µM forward and reverse primer, and 50 ng DNA. PCR reactions were performed on a PTC-200 Peltier Thermal Controller (Biozym, Hessisch Oldendorf, Germany). Amplification was achieved by heating for 10 minutes on 95ºC, followed by 40 cycli of 30 seconds on 95ºC, 45 seconds on 58ºC and 45 seconds on 72ºC. Ten µl PCR product was then mixed with 3 µl loading buffer and run on a 1.5% agarose gel to verify the presence of PCR products of the correct size.

**High resolution melting (HRM) analysis**

HRM analysis of the c.1222C>G and c.912G>T mutations was performed on a Lightcycler 480 System (Roche Applied Science, Mannheim, Germany) in a 96 wells plate by using the HRM Master Mix (Roche Applied Science) according to the manufacturer´s instructions. DNA amounts were 75 ng per sample to which we added 25 ng DNA from a healthy control that had no SNPs according to the dbSNP database build 130 (<http://www.ncbi.nlm.nih.gov/projects/SNP/>). In all plates, one heterozygous and two homozygous mutation carriers were used as positive controls. Amplicons for HRM analysis of both the c.1222C>G and c.912G>T mutations were generated by using DOLK Ex1_4 primers (Table S1) in 35 cycli. In total, 241 healthy controls individuals were tested. Data were assessed by using the LightCycler 480 Gene Scanning Software (Roche Applied Science). All normalized and temperature-shifted plots from positive controls clearly deviated from those of the controls.

HRM analysis of the c.3G>A mutation was performed on a Rotor-Gene Q 2plex HRM system (Qiagen, Venlo, The Netherlands) in a 72 well ring by using the type-it HRM PCR kit (Qiagen) according to the manufacturer´s instructions. Specific HRM primers (Table S1) were designed by the primer3 program (<http://frodo.wi.mit.edu/primer3/>). DNA amounts were 20 ng per sample. In each 72 well ring, DNA from the patient with c.3G>A mutation was used as positive control. In total, 233 healthy controls individuals were tested. Standard HRM software from Qiagen was used to analyse the data. All normalized and temperature-shifted plots from the positive control clearly deviated from those of the controls.

**1000 genomes and Dutch exome sequencing data**

All three *DOLK* mutations were not found in 679 healthy Caucasian controls from the 1000 genomes project [4] and 137 healthy Dutch controls analysed by exome sequencing, which was performed as described previously [5].

**DOLK enzyme analysis**

Skin fibroblasts were cultured in M199 medium (Life Technologies) supplemented with 10% fetal calf serum and 1% penicillin/streptomycin in a humified atmosphere containing 5% CO_2_ at 37 °C. Fibroblast pellets (5 x 10^6^ cells) were resuspended in 175 µl 100 mM Tris-HCl (pH 7.4) and sonicated (3 x 8 seconds), after which Tris-HCl (175 µl, 100 mM containing 0.2 % Triton X-100) was added. Samples were stored at -80 ˚C and sonicated for 3 seconds after thawing before use as crude cell-extract proteins. Protein concentration was determined by Coomassie (Bradford) Protein Assay Kit.

The reaction mixture contained 100 mM Tris-HCl buffer (pH 7.4), 0.1 % Triton X-100, 30 mM CaCl_2_, 20 mM uridine 5’-triphosphate (UTP), 30 µM cytidine 5’-triphosphate (CTP), [γ-^32^P]cytidine 5’-triphosphate (specific activity 6000 Ci/mmol; 1.5 µCi/incubation) and 5 µg dolichol-19 (dolichol in hexane was evaporated under N_2_ and dissolved in Tris-HCl with 0.1 % Triton X-100 with vigorous vortexing).

The incubation (in triplicate) was started by the addition of up to 140 µg of crude cell-extract proteins to a final volume of 100 µl. Blanc incubations (in duplicate) were carried out without dolichol. After incubation for 20 minutes at 37 ˚C, the reaction was stopped by addition of chloroform/methanol (900 µl, 2:1 v/v), mixed and incubated for 30 minutes at room temperature. After addition of 190 µl water and vortexing, the mixture was centrifuged at 500g for 10 minutes. The upper water-containing layer was removed and the remaining organic layer was transferred to a new glass tube, and washed by addition of 900 µl chloroform/methanol/water (3:48:47 v/v/v), vortexed and centrifuged at 500 g for 10 minutes. Washing was repeated twice. After the final washing step, 500 µl chloroform was used for scintillation counting in Ultima Gold XR on a LSC Tricarb (Perkin Elmer).

***DOLK* expression analysis**

SYBR Green-based real-time quantitative PCR (qPCR) expression analysis was performed on a 7500 Fast Real-Time PCR System (Applied Biosystems, Foster City, CA, USA) by using Power SYBR Green PCR Master Mix (Applied Biosystems, Foster City, CA, USA) according to the manufacturer´s instructions. Primers were designed by the primer3 program (<http://frodo.wi.mit.edu/primer3/>). Primer sequences are given in Table S1. *GUSB* and *PPIB* were used as reference genes. Total RNA from different human adult and fetal tissues was ordered from Stratagene Europe (Amsterdam, The Netherlands). All fetal tissues are from 20 or 21 weeks-old embryos after gestation, except for cochlear RNA that was isolated from an 8 weeks-old embryo by using the NucleoSpin RNA II kit (Macherey-Nagel, Düren, Germany) according to the manufacturer’s protocols. To remove residual traces of genomic DNA, the cochlear RNA was treated with DNase I (Invitrogen, Leek, The Netherlands) while bound to the RNA binding column. The integrity of the RNA was assessed on 1.2% agarose gel, and the concentration and purity determined by optical densitometry. The OD_260_/OD_230_ and OD_260_/OD_280_ ratios were in between 1.8 and 2.0. Of all tissues, 5 µg of total RNA was transcribed into cDNA by using the iScript cDNA synthesis kit (Bio-Rad Laboratories, Hercules, CA, USA) according to the manufacturer’s protocol. cDNA was purified by using the NucleoSpin extract II kit (Macherey-Nagel, Düren, Germany) according to the manufacturer’s protocol. qPCR quantifications were performed in duplicate on the equivalent of 12.5 ng total RNA input. Experimental threshold cycles (Ct) values were within the range of cDNA dilutions used to validate the primers. The melt curves of all PCR products showed a single PCR product. All water controls were negative. Differences in expression of a gene of interest between two samples were calculated by the comparative Ct or 2^ΔΔCt^ method [6,7].

**Immunohistochemistry and western blotting**

For immunohistochemistry, fresh heart muscle tissues were frozen in isopentane at -130 ºC and cryosections of 4 µm were cut. After fixation in ice-cold acetone for 10 minutes, the sections were incubated with a 20% solution of normal goat serum (Vector Laboratories) in PBS for 10 minutes. One slide was stained with hematoxylin-eosin for morphological comparison. The sections were incubated overnight at 4ºC with one of the following primary mouse monoclonal antibodies diluted in PBS containing 1% BSA: mouse anti-rabbit alpha-dystroglycan, clone IIH6C4 (Millipore), Mouse anti-human beta-dystroglycan (Novocastra Laboratories), Mouse anti-human beta-sarcoglycan clone/5B1 (Monosan Xtra) or Mouse anti-human desmin clone 33 (BioGenex Laboratories). The sections were washed in PBS and incubated with Alexa Fluor_*_488 Goat anti-mouse IgG (Invitrogen) for 30 minutes. After being washed, the sections were mounted in Vectashield mounting medium for fluorescence (Vector Labatories). The sections were examined with a Leica DM 4000 B fluorescence microscope and pictures were made with a Leica DFC 300 FX camera. Exposure times were equalized for the different stainings.

Western blotting of beta-dystroglycan, laminin overlay and alpha-dystroglycan glycosylation using IIH6 was performed on WGA-enriched heart homogenates as described [8]. One adaption was made: beads were boiled for 15 minutes in loading buffer (10mM Tris, 1mM EDTA, 0.1% SDS, Complete Protease Inhibitor Cocktail from Roche and 4mM DTT).

For western blotting of non-enriched samples, heart muscle (13-14 mg) was solubilized in lysis buffer (100 mg/ml, 125mM Tris, pH 8.8, containing 4% SDS, 40% glycerol, 100mM DTT, Complete Protease Inhibitor Cocktail and bromophenol blue) and incubated at 95⁰C for 15 minutes, followed by 10 minutes at 50⁰C. Homogenates were centrifuged at 13,000 rcf for 5 minutes at room temperature. The supernatant was mixed with sample buffer and used for western blotting. Samples were run on a 7.5% (alpha-dystroglycan, beta-dystroglycan and desmin) or a 12.5% (beta-sarcoglycan) homogeneous gel on a PhastSystem gel electrophoresis system (GE Healthcare) following standard protocols and blotted on nitrocellulose (0.2µm Protran Watman) by diffusion blotting (1 hour at 60^o^C). After blocking (in PBS containing 5% dry milk and 0.5% Tween-20), the membrane was incubated overnight at 4ºC with the following primary antibodies: mouse anti-rabbit alpha-dystroglycan (clone IIH6C4, diluted 1:2500 in PBS containing 1.5% BSA and 0.5% Tween-20), mouse anti-human beta-dystroglycan (diluted 1:250 in PBS containing 1.5% BSA and 0.5% Tween-20), mouse anti-human beta-sarcoglycan (clone/5B1, diluted 1:100 in PBS containing 5% dry milk and 0.5% Tween-20) or mouse anti-human desmin (clone 33, diluted 1:100 in PBS buffer containing 5% dry milk and 0.5% Tween-20). After washing, membranes were incubated with peroxidase-labeled goat-anti-mouse IgG (Santa Cruz, diluted 1:5000 in PBS containing 1.5% BSA and 0.5% Tween-20; alpha-dystroglycan and beta-dystroglycan) or peroxidase-labeled polyclonal goat-anti-mouse IgG (Pierce, diluted 1:500 in PBS containing 1.5% BSA and 0.5% Tween-20; beta-sarcoglycan and desmin) for 2 hours at room temperature. Membranes were developed with ECL using the LAS 3000 detection system (FujiFilm Luminescent Image Analyser). Laminin overlay was performed as described [8].

For CD63 western blotting, fibroblasts (2 x 10^6^ cells) or heart muscle (50 mg in small pieces) were homogenized in lysis buffer (100 µl, 50mM potassium phosphate, pH 6.5, containing 0.5% Triton X-100 and Complete Protease Inhibitor Cocktail) by sonication (2x 10 seconds on ice). Homogenates were left on ice for 15 minutes and centrifuged at 20,000 rcf (20 minutes, 4°C). The supernatant was diluted 1:1 in sample buffer without DTT and used for western blotting. Samples were run on a 12.5% PAA gel on a PhastSystem and blotted on nitrocellulose by diffusion blotting (1 hour at 60^o^C). After blocking (1 hour at room temperature, in PBS containing 5% dry milk and 0.5% Tween-20), the membrane was incubated with primary mouse anti-human CD63 (Developmental Studies Hybridoma Bank, The University of Iowa, Department of Biology, Iowa City) at a 1:2000 dilution in PBS containing 1.5% BSA and 0.5% Tween-20 for 2 hours at RT. After washing, peroxidase-labeled goat-anti-mouse IgG (Dako, Glostrup Denmark) was added at a 1: 5000 dilution in PBS containing 1.5% BSA and 0.5% Tween-20 and incubated for 1 hour at RT. Membranes were developed with ECL reagent (Pierce) using the LAS 3000 detection system (FujiFilm Luminescent Image Analyser).

**References**

1. Rozen S, Skaletsky H (2000) Primer3 on the WWW for general users and for biologist programmers. Methods Mol Biol 132:365-86.

2. Oetting WS, Lee HK, Flanders DJ, Wiesner GL, Sellers TA, et al. (1995) Linkage analysis with multiplexed short tandem repeat polymorphisms using infrared fluorescence and M13 tailed primers. Genomics 30:450-458.

3. Brownstein MJ, Carpten JD, Smith JR (1996) Modulation of non-templated nucleotide addition by Taq DNA polymerase: primer modifications that facilitate genotyping. Biotechniques 20:1004-1010.

4. Durbin RM, Abecasis GR, Altshuler DL, Auton A, Brooks LD, et al. (2010) A map of human genome variation from population-scale sequencing. Nature 467:1061-1073.

5. Vissers LE, de Ligt J, Gilissen C, Janssen I, Steehouwer M, et al. (2010) A de novo paradigm for mental retardation. Nat Genet 42:1109-1112.

6. Livak KJ, Schmittgen TD (2001) Analysis of relative gene expression data using real-time quantitative PCR and the 2(-Delta Delta C(T)) Method. Methods 25:402-408.

7. Pfaffl MW (2001) A new mathematical model for relative quantification in real-time RT-PCR. Nucleic Acids Res 29:e45.

8. Michele DE, Barresi R, Kanagawa M, Saito F, Cohn RD, et al. (2002) Post-translational disruption of dystroglycan-ligand interactions in congenital muscular dystrophies. Nature 418:417-422.

9. Kranz C, Jungeblut C, Denecke J, Erlekotte A, Sohlbach C, et al. (2007) A defect in dolichol phosphate biosynthesis causes a new inherited disorder with death in early infancy. Am J Hum Genet 80:433-440.

10. Haeuptle MA, Hennet T (2009) Congenital disorders of glycosylation: an update on defects affecting the biosynthesis of dolichol-linked oligosaccharides. Hum Mutat 30:1628-1641.
